# The Clinical Significance of Unknown Sequence Variants in *BRCA* Genes

**DOI:** 10.3390/cancers2031644

**Published:** 2010-09-10

**Authors:** Valentina Calò, Loredana Bruno, Laura La Paglia, Marco Perez, Naomi Margarese, Francesca Di Gaudio, Antonio Russo

**Affiliations:** 1 Department of Surgery and Oncology, Regional Reference Center for the Biomolecular Characterization and Genetic Screening of Hereditary Tumors, University of Palermo, Via del Vespro 127, 90127 Palermo, Italy; E-Mails: valevicio@yahoo.it (V.C.); loredana.bruno@unipa.it (L.B.); l.lapaglia@libero.it (L.L.P.); marcoperez@inwind.it (M.P.); m.naomi84@gmail.com (N.M.); 2 Department of Medical Biotechnologies and Legal Medicine, University of Palermo, Palermo, Italy; E-Mail: francescadigaudio@unipa.it (F.D.G.)

**Keywords:** BRCA genes, variant, integrated models, oncogenetic counseling

## Abstract

Germline mutations in *BRCA1/2* genes are responsible for a large proportion of hereditary breast and/or ovarian cancers. Many highly penetrant predisposition alleles have been identified and include frameshift or nonsense mutations that lead to the translation of a truncated protein. Other alleles contain missense mutations, which result in amino acid substitution and intronic variants with splicing effect. The discovery of variants of uncertain/unclassified significance (VUS) is a result that can complicate rather than improve the risk assessment process. VUSs are mainly missense mutations, but also include a number of intronic variants and in-frame deletions and insertions. Over 2,000 unique *BRCA1* and *BRCA2* missense variants have been identified, located throughout the whole gene (Breast Cancer Information Core Database (BIC database)). Up to 10–20% of the *BRCA* tests report the identification of a variant of uncertain significance. There are many methods to discriminate deleterious/high-risk from neutral/low-risk unclassified variants (*i.e*., analysis of the cosegregation in families of the VUS, measure of the influence of the VUSs on the wild-type protein activity, comparison of sequence conservation across multiple species), but only an integrated analysis of these methods can contribute to a real interpretation of the functional and clinical role of the discussed variants. The aim of our manuscript is to review the studies on BRCA VUS in order to clarify their clinical relevance.

## 1. Introduction

The identification of high penetrance alleles of the *BRCA1* (MIM 113705) and *BRCA2* (MIM 600185) genes, which determine a high risk of developing hereditary breast and ovarian cancer, has made genetic testing an integral part of oncogenetic counseling in clinical practice [1,2,3,4,5,6].

Such germline mutations lead to a risk of developing breast cancer of 45% at the age of 70 in *BRCA1* carrier women and the risk of an ovarian neoplasia of about 40% at age 70, whereas in *BRCA2* mutation carriers breast cancer risk is of 35% at age 70 and about 20% of ovarian cancer at age 70 [7].

The lack of mutational hotspots leads to the analysis of the entire coding sequence of the *BRCA* genes in the various hospital, research and private clinically certified genetic testing laboratories involved, leading to the identification of thousands of different mutations associated with the disease.

The Breast Cancer Information Core Database (BIC database), a public archive of *BRCA* mutations, has collected the whole series of *BRCA1* and *BRCA2* coding variants, amounting up until now to about 1,300 deleterious mutations in the two genes, identified from various population studies (Table 1).

**Table 1:** International sites for variants of uncertain/unclassified significance (VUS) studies.

	**Mutation nomenclature**		http://www.hgvs.org/mutnomen/	
			http://www.humgen.nl/mutalyzer/1.0.1/http://research.nhgri.nih.gov/bic	
	**Co-occurence in-trans**		www.dmubd.net	
	**Species conservation**		www.agvgd.iarc.fr/index.php	
			www.ebi.ac.uk/clustalw/index.html	
	***In silico* analysis**		http://www.russell.embl.de/aas/	
			http://coot.embl.de/PolyPhen	
			http://www.agvgd.larc.fr	
			http://blocks.fhcrc.org/sift/SIFT.html	
			http://genetics.bwh.harvard.edu/pph/	
			http://www.ensembl.org/index.html	
			www.uniprot.org/	
			www.expasy.ch/prosite/	
	**Splice-site prediction**	Berkley Drosophila Genome project	www.fruitfly.org/seq_tools/splice.html	
		NetGene2	www.cbs.dtu.dk/services/NetGene2	
		Alex Dong Li's splice site finder	www.genet.sickkids.on.ca/~ali/splicesitefinder.html	
		GeneSplicer Web Interface	www.tigr.org/tdb/GeneSplicer/gene_spl.html	
		Splice Sequence Finder(Montpelier)	www.umd.be/SSF	
	**SNP Database**		www.ncbi.nlm.nih.gov/proiects/SNP/	

About 70–80% of the mutations identified in the *BRCA* genes are pathological, as they produce a non-functional protein. Such mutations give rise to the premature introduction of stop codons due either to small insertions or deletions, which result in a frameshift in the reading frame, or else to nonsense mutations or to alterations in the exon-intron boundaries, which lead to incorrect RNA transcripts. Only 6% of the missense mutations included in the BIC database are deleterious due to leading to a change in the amino acid residues of the functional motives of the BRCA protein.

One percent of all the coding variants are due to large deletions or duplications in the *BRCA* genes. All these pathological variants lead to a *positive* genetic test, involving the identification of a mutation which clearly destroys the function of the protein and therefore permits physicians to program the test for either healthy or affected relatives, but above all, make it possible to plan prevention strategies.

Other possible results emerging from sequence-based genetic tests are *negative—*where no coding variant is analyzed; or *uncertain*—when a VUS provides no information concerning the function of the gene and therefore offers no information regarding cancer risk.

The main problem for physicians is the interpretation of the VUS results, which up to the present day amount to over 1,500 [8,9,10]. 

During the last few years, several approaches have been proposed for the evaluation of the clinical relevance of these VUSs. The study and interpretation of new or rare variants involves, in fact, integrated studies of several different types of analyses, such as co-segregation with the disease, concurrence with a deleterious *in trans* mutation, personal and family history of cancer of the carrier, *in silico* assessment of phylogenetic conservation and severity of the protein modification in biochemical functional assays [9,11,12,13,14]. 

First of all, these studies help to complete and clarify the involvement of a variant at the onset of the proband’s disease and then make it possible to attribute a high or low risk of development of breast and/or ovarian disease to the VUS carriers within the family. 

Deleterious changes increase cancer risk dramatically, whereas neutral polymorphic changes do not seem to.

The aim of this review is to help physicians to reach a clearer understanding of the number and type of the parameters that may lead to a classification of the risk level in subjects who are carriers of VUS, in order to avoid personalized surveillance programs based on family history, focusing rather on more appropriate intervention strategies and prevention programs.

## 2. Variants of Unknown Significance (VUSs)

VUSs are mainly missense and splice site mutations that have no clear biological relevance. This group may also include intronic variant mutations, small in-frame insertions and deletions, and nucleotide substitutions that do not result in an amino acid change (silent variant). These mutations together constitute about 30% of all the variants reported in the BIC database, and their effect on the protein function is not clear causing a difficult classification and interpretation as deleterious *versus* neutral polymorphic classes. 

The Myriad Genetic Laboratories (Salt Lake City, UT) report that about 7% of their molecular diagnoses of hereditary breast/ovarian cancer are linked to VUS [8,9]. These alterations have been identified more commonly in the Afro-American than in the Hispanic population [15,16].

It is clear that is very important to understand and interpret the functional significance of such variants.

The first studies regarding the interpretation of the clinical significance of VUSs in *BRCA1* date to Judkins *et al.*, who tried to define a first and global technical approach for this [17]. In recent years, the same approach has been used for the interpretation of VUSs found in the *BRCA2* gene [17,18].

Actually, one of the most active research groups in this field is the IARC Unclassified Genetic Variants Working Group, which aims at collecting as much information as possible from the various population groups with the use of database and programming facilities [14,19,20,21,22,23,24]. 

## 3. Principal Methods of VUS Assessment

### 3.1. Type and Location of VUSs

When a missense VUS is reported, primarily it has to be identified whether the amino acid change is located in a functional domain of the protein. For example, BRCA1 has a well-defined RING finger domain at the *N*-terminus and BRCT repeats at the C-terminus of the protein. Many missense VUSs occur within the RING-finger domain of the protein and these will most probably give rise to a functional loss.

On the contrary, it is more difficult to assess a pathological effect for variants in the exon-intron boundaries, due to their involvement in the splicing process. Analytical studies of the transcript product are required in order to define their effect [25]. 

Mutations identified in such well conserved functional domains are considered as suspected deleterious, *i.e.* the variants for which the available evidence indicates the likelihood, but not definite proof, that the mutation is deleterious. Such alterations require further supporting steps in the study of missense VUSs, such as the amino acid conservation analysis in the species and segregation analysis in breast and ovarian cancer families. 

In 2006, Bonatti *et al.* studied six splice variants, five of which caused a completed inactivation of the mutant allele since they introduced a frameshift. The aberrant RNA splicing was identified in the probands carrying genomic variants by the BDGP (Berkeley Drosophila Genome Project) computer program that allows prediction of the effects of a variant on splicing efficiency by comparing the sequence containing the consensus splice site *versus* the sequence variant (Table 1).

It has to be considered that the aberrant RNA splicing has to be supported by other analyses such as the genotype-phenotype correlation and the cosegregation analysis in healthy relatives, which confirms the pathogenicity of alleles with splicing site mutations [26].

Finally, the functional and clinical consequences of the splicing process can be assessed by a variety of bioinformatic prediction programs, by *in vitro* analysis of the mRNA of the lymphocytes in normal tissue and by transcript analysis carrying a premature stop codon or in frame deletion disrupting a known functional domain [23]. 

Following the analysis of mutation type, the next step involves consultation of the relevant literature and the various mutation and SNP databases (Table 1).

The BIC database has recently added the possible clinical significance of the variants to the description of the identified mutations, to give the opportunity to be studied by all researchers that do population studies. 

### 3.2. Analysis of Control Group

*BRCA* gene mutations have been identified according to the ethnic group and geographical area; a useful method for the study of VUSs therefore involves the analysis of the variant in a control group [27,28,29]. When this variant in a control group has an allele frequency of more than 2%, it is to be considered a polymorphic variant with a neutral clinical significance.

It is important to consider both size and ethnic homogeneity, since the research of the Myriad Genetic Laboratories involves subjects with European ancestry and cannot therefore be used for the study of a genetic variant occurring in a patient with a different (*i.e.*, Asian) ancestry [30].

### 3.3. Co-Segregation with Cancer in Families

Co-segregation analysis allows the association of each variant with disease. It may support the study of the clinical significance both of missense VUSs and also of those found within the non-coding portions of the *BRCA* genes. 

The identification of the variant in family members with the disease but not in healthy members, presupposes that the involved variant is pathological [7,11].

In fact, as reported by Goldgar *et al*., the co-segregation with disease in pedigrees provides evidence for the potential use for co-segregation analysis for VUS classification, as it is easily quantifiable and directly related to disease risk. In addition, this method is not susceptible to uncertainties in mutation frequencies or population stratification. 

Unfortunately, the main problem of the segregation study regards the availability of family data and its size, requiring sampling of additional individuals in the pedigrees (particularly additional cases), which may be difficult to achieve [7,11]. 

### 3.4. Co-occurrence with Known Deleterious Mutation

Co-occurrence data are relatively easily available; however, when applied to the actual set of unclassified variants, this analysis has much better power to contribute to classification of recurrent neutral variants than to classification of recurrent deleterious variants.

Because homozygous *BRCA1* mutations are expected to be lethal and homozygous *BRCA2* mutations are expected to result in lethality or a phenotype such as Fanconi Anemia, the presence of a deleterious mutation *in trans* with a variant suggests its neutrality [31,32,33]. 

In 2003, Judkins *et al.* found over 500 genetic variants that were successfully assigned to one or more haplotypes using an automated computer program [34]. The haplotype assignments were used to identify 20 different VUSs in patients *in trans* with known deleterious mutations, confirming them as neutral.

### 3.5. Conservation of Amino Acids across Species

A comparative analysis of amino acid conservation from multiple species by protein sequence alignments provides an understanding of the importance of missense VUSs in the different *BRCA* protein domains [35,36]. The alignment of the orthologous sequences of several domains of the BRCA1 protein provide an indication of which amino acid residues are truly conserved and which of them represent localized evolution. The alteration of these residues probably leads to a pathogenic sequence variant and the different data combinations of the Bayesian analysis permits an evaluation of the evolutionary significance of the missense variant involved. When the domains are not conserved, the *in silico* analysis of the variant is required [37]. Although this approach should be applied to all domains of the BRCA proteins, it acquires more relevance according to the function of the domain analyzed. Three main conserved domains are studied; these are the BRCA1 RING-domain (amino acids 1 to 102), the BRCA1 C-terminus domain (amino acids 1641 to 1863) and the BRCA2 DNA binding domain (amino acids 2401 to 3200) [38].

### 3.6. In silico Analysis of Amino Acid Change

The analysis of the consequence of an amino acid change on the protein function is extremely important and is often used for missense VUSs.

*In silico* analysis of the chemical nature of the amino acid residues can be performed by computer programs, such as the Grantham chemical difference matrix which analyzes the relationship of one amino acid residue to another. This relationship includes side chain composition, polarity and steric features and implicates the insertion of the sequence variant leading to the modification of the protein structure.

As Grantham reports, its algorithm is based on a formula regarding the difference between amino acids, combining properties that correlate best with protein residue substitution frequencies. This takes into account chemical and physical characteristics between amino acids, and compares these differences between exchanging residues with substitution frequencies. Correlation coefficients show that fixation of mutations between dissimilar amino acids is generally rare [39]. 

Extending the Grantham difference to a two-variable system (GV and GD) improves the ability to distinguish between substitutions that are likely to be pathogenic and those that are not. The Alignment GVGD score prediction analysis combines together an extensive protein multiple sequence alignment based on quantitative measures of sequence variation (Grantham Variation or GV) and quantitative measure of the fit between a missense substitution and variation observed at its position in the protein multiple sequence alignment (Grantham Deviation or GD) [33]. 

This so-called Align-GVGD (Grantham Variation/Grantham Deviation) is otherwise a mathematically simple classification of missense substitutions in breast cancer susceptibility genes, providing a class probability based on evolutionary conservation and properties of the amino acid (Table 1) [9]. Nevertheless, the distribution of genetic risk as a function of this Align-GVGD is still not fully understood [24]. 

### 3.7. Loss of Heterozygosity (LOH)

*BRCA* mutation-positive tumors present loss of heterozygosity (LOH) more frequently than sporadic forms. This feature can be used to assess the pathogenicity of *BRCA* VUSs [12,40,41]. The unidentified variants in the tissue will probably prove to be neutral.

This method is also often associated with the family data in order to support the hypothesis of pathogenicity or neutrality, but this requires the availability of tumor tissue from the *BRCA* carriers.

Allele-specific LOH studies have been conducted on carriers of known deleterious mutations and high frequency rates of *BRCA* LOH for breast and ovarian tumors were found.

This study could be useful to classify *BRCA* VUS if associated and incorporated with integrated models. 

### 3.8. Biochemical Functional Assays

Functional assays that are able to measure the influence of variants on protein activity can only be established following development of a detailed understanding of how a protein functions in a cell and how it contributes to cancer predisposition when mutated. 

Since the complete and effective BRCA protein function is still not fully understood, it is unknown which *in vitro* method should be used in order to define the pathogenicity or neutrality of a VUS.

Recently, different methods have been developed to analyze *BRCA1* gene mutations, such as the transcriptional and BARD1-correlated ubiquitin ligase activity, and those regarding the *BRCA2* gene mutations involving homologous recombination repair.

#### 3.8.1. Protein Assays Related to BRCA1 Function

The BRCA1 protein has an ubiquitin ligase activity BARD1 mediated through its RING-finger domain [42,43]. Pathogenic mutations have already been identified in this domain and have been correlated with the loss of this ligase activity [44]. This observation has led to the development of various methods aimed at evaluating the functional effect on the protein interaction between BRCA1 and BARD1 and UbcH5a (enzyme conjugated to Ubiquitin E2), respectively. 

Actually, of the 100 variants identified in this interaction domain, only those which completely annul the interaction with BARD1 and with UbcH5a, resulting in the loss of the ubiquitin ligase activity, are classified as pathogenic [45].

Transcriptional activity located in the C-terminus region has been evaluated by the DNA Binding Domain (DBD) fusion method in yeast and mammalian cells, using expression vector constructs coding for a DNA binding domain fused to residue 1396–1863 of the gene [14,46,47].

Nevertheless, although this method has been used to evaluate over 50 VUSs from the BRCA1 BRCT domains, this analysis is limited to the C-terminus region. 

#### 3.8.2. Protein Assays Related to BRCA2 Function

The BRCA2 protein has a BRCT functional domain, which enables it to function in homologous recombination repair of double strand DNA breaks and to associate with RAD51. The BRCA2 homologous recombination repair assay can be measured by a Green fluorescent protein (GFP)-dependent homology directed assay method in mutated cell lines altered in the DNA repair system [14,48]. About 30 variants of *BRCA2* have been studied with this method [12,18,49]. This method can also be applied to other variants of BRCA2 and to other domains, such as those involving the RAD 51 binding or the interaction domain with PALB2. However, there is still no clear understanding regarding the correlation between the method and the risk of cancer associated with mutations in this region, where pathogenic missense mutations leading to an increase in breast and ovarian cancer risk have not yet been identified [50]. 

It is clear that biochemical and functional studies of important protein domains cannot be considered singularly, but must be associated with multifactorial models. Nevertheless, there exists a strong correlation between the two types of analysis, indicating high sensitivity and specificity of the methods [18]. In fact, these assays may be considered as part of a multivariate model for the evaluation of VUS carriers. 

This is an extremely efficient approach for the classification of VUSs, which is able to differentiate between those that inactivate or have no influence on BRCA2 function; it can only be used, however, if data regarding the family data are available.

### 3.9. Pathological Data

A variety of experimental and statistical approaches for the classification of *BRCA1* and *BRCA2* variants has already been mentioned. Recently, however, there has been a growing interest in developing additional efficient and reliable ways for classifying VUSs (*i.e.*, histopathological and immunohistochemical profiles).

*BRCA1*- and *BRCA2*-related tumors develop largely through distinct genetic pathways in terms of the regions altered, while also displaying distinct phenotypes. In particular, *BRCA1*-associated breast cancer (BC) is more often estrogen receptor (ER), progesterone receptor (PgR) and HER2neu receptor negative (triple negative), is more often of the basal-like and medullary phenotype, and more frequently has a high mitotic count and histological grade [21,38,51,52].

Breast cancer due to a *BRCA2* mutation is also of a higher histological grade than sporadic breast cancer, and it displays luminal phenotypes and rarely overexpresses *HER-2* gene products, although the difference is less pronounced as compared with *BRCA1*-related tumors [53,54].

It is therefore important to conduct further analysis on VUS carriers, in order to reach a more detailed classification and to see if they show similar features to *BRCA*-related carriers. 

## 4. Integrated Models for VUS Assessment

The previously mentioned factors (LOH, co-occurrence with a known deleterious mutation *in trans*, sequence conservation or splice site analysis, pathological data and personal cancer history) are defined as independent variables, as they allow only partial evaluation of the clinical significance of variants. Often these data do not include segregation analysis or familial information, which are more difficult to acquire. 

Different researchers have tried to use direct and indirect epidemiological observations to clarify the power and the influence of the VUS as high- or low-risk categories, since the tested individuals and their families, may provide a seemingly ambiguous result, unless sufficient evidence is available that a given missense change is deleterious [11,37,55,56]. 

Each of these methods of analysis and interpretation of results have strengths and limitations, as the frequency of mutations in cases and controls provides a direct estimate of associated cancer risk, but as those variants are presented rarely, such studies would need a very large cohort study. Species conservation and analysis of amino acid changes do not require the information of many families, but is limited by the fact that it is only indirectly related to the cancer risk and need to be associated with other additional information or analysis methods. 

A method proposed to help physicians to interpret and classify VUSs is the *Polyphen*-based classification. This is an algorithm which classifies VUSs based on the functional effect of each missense variant into three categories (probably damaging or deleterious, possibly damaging, and benign) (Table 1). This model makes use of the chemical characteristics of the substitution site, the alignment of homologous sequences and protein three-dimensional structures, but is limited to the classification of missense variants, leaving unresolved the problem of how to interpret other types of VUSs that are not missense. 

It is clear that a combined model involving all possible direct and indirect, quantitative and qualitative observations is needed to give a more specific definition for each variant in terms of pathogenicity. 

Over the last several years many researchers have devised algorithms for *in silico* assessment of missense substitutions [9,12,37,57,58].

Among these *in silico* analyses, the Grantham difference has been a popular source of data, contributing to the assessment of the pathogenicity of missense substitutions [37,39,59].

As previously reported, this model has more recently been revised by Tavtigian, extending it to the two-variable system A-GVGD, improving the sensitivity and power of prediction of the previous method [33]. 

In 2008, Tavtigian *et al.* published an interesting study proposing an alternative and extended multifactorial likelihood model for classification of VUSs that adds two other measures to the Alignment GVGD that can contribute to a better estimation of the genetic risk: the ascertainment ratio (AR) and the enrichment ratio for single-nucleotide substitutions (ERS) [33,59]. The AR is a measure of genetic risk closely related to an odds ratio, but designed to be applied to aggregated pools of rare sequence variants. The ERS is closely related to the codon-based measure of evolutionary selective pressure [60]. This multifactorial approach tries finally to order more specifically VUS by genetic risk. 

Recently Goldgad *et al.* proposed another integrated model to classify VUSs based on a combination of variables from the cancer history of the probands and their families that significantly distinguish families with proven deleterious variants from those with neutral variants [11]. This model takes account of direct epidemiological data, including cosegregation analysis, case-control analysis, personal and family history and co-occurrence of mutation disease; together with indirect measures, including amino acid conservation, severity of amino acid change, and evidence from functional assays. The model initially proposed has been revised, including other statistical studies [19,61,62]. We must consider that it is difficult to find a model that includes all variables and at the same time analyze the relationships among the different forms of evidence and integrate them to form one conclusion.

Osorio *et al.* [38] have developed a likelihood-based model, integrating most of the data currently used to classify these variants including LOH, grade, and immunohistochemical analysis to assess estrogen receptor status for the tumors of carriers of these variants. They also considered the summary family history (personal or first-degree family history of bilateral breast or ovarian cancer), previously proposed by Goldgar [19].

The result of this latter study indicates that VUS carriers are characterized by ER negative expression and histological grade 3. Moreover, the information regarding LOH, ER status, grade, and personal and first-degree family history from multiple tumors of carriers of BRCA1 missense VUSs can be sufficient to estimate odds of causation of an adequate order to classify them as neutral or deleterious. Immunohistochemical profile, strong family history and early age onset were used by Sweet *et al.* to classify L22S and T37K as deleterious BRCA1 variants [25].

**Figure 1:**
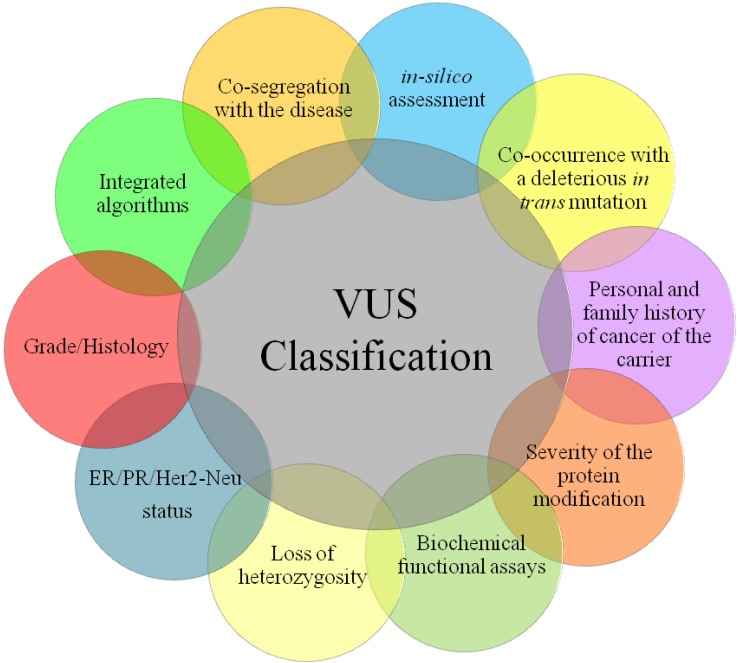
A representation of the multi-determinant approach to use for *BRCA*-VUS clinical classification.

All these integrated models are principally used for the analysis of missense variants, thus still leaving open the debate on the best choice for the characterization of other variants of unknown significance such as *splice sites* and *in-frame* variants. The only methods applied nowadays for this aim are *in vitro* and *in silico* analyses, but it is necessary to find an integrated method or models to combine with these (Figure 1). 

## 5. Psychological Aspects of VUS Carriers

The VUS category has always posed many problems in genetic counseling due to the difficulty in interpreting the clinical significance of the new or rare variants [63]. 

This difficulty is not only to be seen strictly from a molecular and genetic point of view, but giving an ambiguous result for tested individuals means that the clinician cannot manage the problem, probably proposing a wrong follow-up strategy. Moreover the family VUS carriers can develop stress that is not necessarily appropriate for the type and power of the discussed variant.

It is clear that the classification of variants in breast cancer predisposing genes has many benefits for genetic counseling, Women found to carry a pathogenetic variant can be advised to undergo regular screening by mammography or magnetic resonance imaging and to consider risk-reducing surgery [64,65]. On the other hand, relatives who do not carry a pathogenic variant can be reassured that they are not at high risk. 

## 6. Classification System of VUSs

The misinterpretation of VUSs results in inappropriate clinical consequences and establishes confusion among clinicians, who are unable to offer a clear explanation of the risks to the patients involved. A classification system for variants with clinical recommendations would help clinicians on risk prediction, carrier testing, and reproductive decision making, thus reducing the level of misinformation given to the patients.

Many groups are cautious in classifying the variants as pathogenic, especially when there is a lack of support from epidemiological data regarding cancer association.

The BIC database uses a triple classification of clinical importance—“yes, no or unknown”; the latter includes all the variants ranging from a pathogenicity of 0.1 to 99%.

In 2008, Plon *et al.* [22] suggested a standardized classification system for application to sequence-based results, using a Bayesian system to generate a probability which should be the standard for all variants. This classification system involves five classes of variants, with each class given specific recommendations for clinical management of at-risk relatives.

Since Class 1 does not include pathogenic variants, carriers are not required to undergo increased surveillance nor testing of other members of the family, unlike the situation regarding Class 5, which includes pathogenic variants.

Class 2 variants (0.1–5% likelihood of pathogenicity) have the same recommendations of variants as Class 1; these may be considered as uncertain and will therefore be included in Class 3 when there are more data favoring pathogenicity.

**Figure 2:**
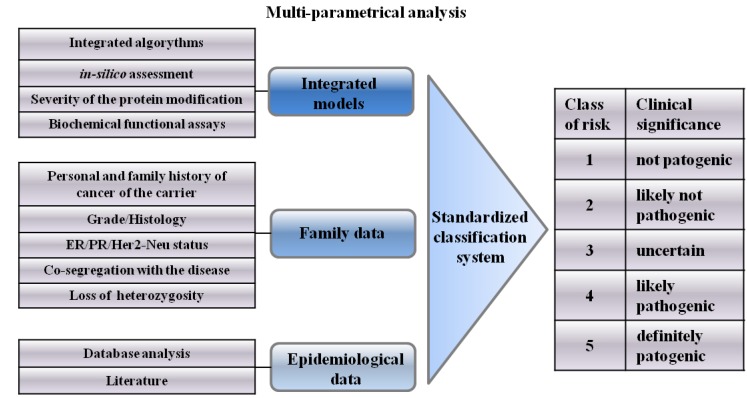
A schematic representation of a multi-parametrical analysis method proposed to help clinicians to individuate the classes of risk.

The most important distinction will thus be between Class 3 and Class 4 variants, since the “uncertain” variant may easily become “probably pathogenic” if there are sufficient relevant family data and segregation analyses [22] (Figure 2). In such cases, it might help to make a further classification of the variant and follow full high-risk surveillance guidelines [64,65].

## 7. Conclusions

VUS classification has posed many problems in genetic counseling due to the difficulty in the interpretation of the clinical significance of the new or rare variants of *BRCA* genes. The tested individuals and their families, in fact, present ambiguous results. 

This review describes both the different approaches and the problems to be faced in the clarification of the pathogenic role of VUSs. 

Different study strategies are necessary for each variant in the attempt to give a clinical interpretation of the test results. Multidisciplinary approaches involving direct and indirect epidemiological observations have been integrated with statistical, mathematical and physical models by Goldgar *et al.* [11] and then revised by many researchers.

Nevertheless, while it is important that analysis methods should be integrated, all the necessary data are not always available, and a single functional assay, or a set of assays, have so far not proved sufficient for the prediction of pathogenicity with absolute certainty.

The future international use of a standardized system of VUS classification will make it possible to guarantee the clinical usefulness of genetic testing and an unequivocal clinical method for the treatment of at-risk patients. The most important prospective should be the creation of a unique database for the calculation of the likelihood of the pathogenicity of variants, such as the BIC database, which includes all variants in *BRCA* genes identified in different population studies. 
